# Mushroom cultivation in the circular economy

**DOI:** 10.1007/s00253-018-9226-8

**Published:** 2018-07-19

**Authors:** Daniel Grimm, Han A. B. Wösten

**Affiliations:** Microbiology, Department of Biology, Utrecht University, Padualaan 8, 3584 CH Utrecht, The Netherlands

**Keywords:** Mushroom, Edible mushroom, Fungus, Spent mushroom substrate, Circular economy, Mycelium material

## Abstract

Commercial mushrooms are produced on lignocellulose such as straw, saw dust, and wood chips. As such, mushroom-forming fungi convert low-quality waste streams into high-quality food. Spent mushroom substrate (SMS) is usually considered a waste product. This review discusses the applications of SMS to promote the transition to a circular economy. SMS can be used as compost, as a substrate for other mushroom-forming fungi, as animal feed, to promote health of animals, and to produce packaging and construction materials, biofuels, and enzymes. This range of applications can make agricultural production more sustainable and efficient, especially if the CO_2_ emission and heat from mushroom cultivation can be used to promote plant growth in greenhouses.

## Introduction

The transition to a circular economy has shifted from a vision (Boulding 1966) to actual policy making. In this view, agricultural waste streams are no longer considered a debit entry but are considered valuable resources. The 0.25 billion tons of straw that were burned in China alone in 2009 (Feng et al. 2011) could have been used in a wide variety of applications. For instance, lignocellulosic waste streams can be converted into second-generation biofuels. As such, it can contribute to the aim of the European Union to have 10% of the transport fuel originating from renewable sources by 2020 (www.ec.europa.eu/energy/en/topics/renewable-energy). Although use of resources for production of second-generation biofuels does not compete with food, this may not be the most circular application. Growing mushroom-forming fungi on these substrates may prove more sustainable. This would not only result in edible and/or medicinal mushrooms but also in spent mushroom substrate (SMS) that can be used for a wide variety of applications.

Mushrooms represented a market of 63 billion US dollars in 2013 (Royse et al. 2017). This market represents medicinal mushrooms (38%) and wild (8%) and cultivated edible (54%) mushrooms. At a global scale, consumption of mushrooms has increased from 1 to 4.7 kg of cultivated edible mushrooms per capita in the period 1997 to 2013 (Royse et al. 2017). Consumption is expected to further increase in the next years resulting in a sales going from 34 to 60 billion US dollar annually (see e.g., https://www.zionmarketresearch.com/news/global-mushroom-market). In 2013, China produced 87% of the 35 billion kg of cultivated edible mushrooms, most of which being consumed in this country. This explains why the button mushroom (*Agaricus bisporus* and relatives), the most popular edible mushroom in the Western world, is only at the fourth position of most cultivated mushrooms. The top three consists of *Lentinula* (shiitake and relatives), *Pleurotus* (oyster mushrooms), and *Auricularia* (wood ear mushrooms). Edible mushrooms are considered nutritious foods. They contain 5–15% dry matter, have a balanced composition of minerals and vitamins, and are rich in fiber and protein (± 2% fresh weight) (Mattila et al. 2002). Their amino acid composition is better when compared to that of vegetables like potatoes and carrots. Moreover, mushrooms are low in calories (27–30 kcal/100 g) with a low amount of fat (1.3–8% of dry weight mushrooms) and digestible carbohydrate (Mattila et al. 2002).

SMS is available in huge amounts underlined by the fact that 1 kg of fresh mushrooms results in 5 kg of spent substrate (i.e., 2 kg dry weight) (Finney et al. 2009). SMS was long considered a waste stream. Yet, it can be used for production to produce high-quality compost (Uzun 2004; Polat et al. 2009) or other mushrooms (Stamets 1993), to feed animals and to improve their health (Song et al. 2007; Nasehi et al. 2017), to make biofuel production more effectively (Phan and Sabaratnam 2012), to produce materials (Jones et al. 2017; Islam et al. 2017; Appels et al. 2018), and to extract enzymes for industries and bioremediation (Phan and Sabaratnam 2012). In this review, we will discuss production of mushrooms and the potential applications of SMS in a circular economy. We will not discuss the use in bioremediation. For this, we refer to, for instance, Frutos et al. (2016); Siracusa et al. (2017); and Mir-Tutusaus et al. (2018).

## Mushroom production

Cultivated edible mushrooms are the fruiting bodies of basidiomycetes with a saprobic life style. These basidiomycetes can be divided into primary, secondary, and tertiary decomposers (Rahi et al. 2009). Primary decomposers such as the oyster mushrooms (*Pleurotus* spp.) and shiitake (*Lentinula edodes*) degrade (hemi)cellulose, lignin, and other components of plant material. Unlike secondary and tertiary decomposers, they do not depend on other organisms and their metabolites. Secondary decomposers such as the button mushroom typically colonize composted materials, while tertiary decomposers such as *Agrocybe* spp. are generally found in soils. The three categories of decomposers represent a continuum in the metabolic transition from lignocellulosic and other organic materials to soil. Indeed, it is possible to completely compost agricultural waste through the successive cultivation of mushrooms from different stages in this continuum (Stamets 1993). This, however, is hardly, if at all, applied in large-scale mushroom production.

Many mushroom-forming fungi belonging to the class of primary decomposers can be cultivated on a range of lignocellulosic material (Stamets 1993), including various types of straw, cotton seed hulls, corn cobs, peanut shells, cotton from textile industry, coffee pulp, paper (Sánchez 2010), and leaves (Shah et al. 2004). The oldest form of mushroom cultivation is probably the outdoor log culture, which has been used in China to cultivate shiitake at least for a millennium. Nowadays, this technique has largely been replaced with the more effective indoor cultivation on “artificial logs,” plastic bags filled with nutrient complemented sawdust-based substrates. Once the bag is colonized, it is unpacked to allow fruiting. The sawdust is being held together by mycelium, like glue, and will not fall apart. Very similar to the artificial logs are the column cultures that consist of long plastic bags that are hung from the ceiling. Once the mycelium has colonized these bags, holes are punched into the plastic to allow mushroom fruiting. Cultivation of *Pleurotus ostreatus* results in about 50% carbon dioxide, 20% water, 10% mushrooms, and 20% residual compost (SMS) (Stamets 1993). Indeed, a 2:1 ratio of dry SMS (including the vegetative mycelium) to fresh mushroom is a rule of thumb in mushroom cultivation. The substrate composition however will play a large role in colonization and fruiting efficiency. For instance, *Pleurotus florida* colonization results in a dry weight reduction of pea and rice straw of 20 and 12%, respectively (Nasehi et al. 2017).

*A. bisporus* is considered a secondary decomposer. However, Till (1962) demonstrated that *A. bisporus* can be cultivated on non-composted substrates like autoclaved sawdust. Indeed, button mushrooms can be produced on self-pasteurized substrates like corn-cob, primavera tree, and Pangola grass (Colmenares-Cruz et al. 2017) with highest production levels on the latter substrate with a biological efficiency (BE) of 52% and a yield of 7.6 kg m^−2^. After supplementation, yields can be increased to 26 kg m^−2^. This represents a BE of up to 176% and is commercially viable. Still so far, button mushrooms are produced on compost that has undergone a two-phase fermentation process and that is topped with a casing layer of for instance peat. Casing improves water availability (Royse and Beelman 2007), and its bacterial activity likely removes *A. bisporus* volatiles that suppress fruiting (Noble et al. 2009). The components for the compost consist of complex structure and decomposition material (straw, sugarcane bagasse, and/or animal litter), compost activator materials (e.g. urea, soy bran, cottonseeds), and inorganic conditioners (gypsum and lime) (Miller and Maculey 1989). The basis for substrate-formulation depends on local availability of substrates but often a combination of straw and animal manure is used (Royse and Beelman 2007). Phase I of composting takes 3–6 days during which temperature increases to 80 °C due to microbial activity. The metabolic activity of the (thermophilic) microflora helps to create a more selective substrate for *A. bisporus*. Temperature of the compost during phase II is initially 50 °C, followed by a 2-day period at 60 °C and a 3-day period at 45 °C (Gerrits 1988). This temperature regime impacts the composition of the microflora and results in a decrease in ammonium levels in the substrate.

The conversion of horse manure-based substrate during composting and vegetative growth of *A. bisporus* has been studied in detail (Jurak et al. 2015b)*.* Dry mass of the substrate is reduced by 8% in phase I of composting, by another 15% in phase II, ultimately resulting in a loss of 31% after 16 days of vegetative growth of *A. bisporus* (i.e., end phase III). The ash content (mineral fraction) rises from about 21% (*w*/*w* based on dry mass) to 29 and 30% by the end of phase II and phase III, respectively. Nitrogen content rises from 1.3 to 1.4% in phase I and increases further to 2.1 and 2.2% at the end of phase II and phase III, respectively. The amount of nitrogen fixed in protein rises significantly during these phases (i.e., from 0.8% (end phase I) to 1.6% (end phase 2) to 2.1% (end of phase III). The carbohydrate content is hardly affected during phase I and decreases from 44 to 26% during phase II. This reduction is accompanied by a loss of 50–60% of xylan and cellulose (Jurak 2015; Jurak et al. 2015b). In phase III, 50% of the lignin is degraded with an additional decomposition of 15% of the xylan and 10% of the cellulose (Jurak 2015; Jurak et al. 2015b). Experimental evidence suggests that *A. bisporus* feeds on lignocellulose indirectly (Vos et al. 2017a). It was proposed that the breakdown products resulting from the lignocellulolytic enzymes of *A. bisporus* are used by bacteria to support their growth, after which *A. bisporus* feeds on the bacterial biomass. This strategy would alleviate the deficiency of *A. bisporus* to produce molecules such as vitamins.

The vegetative mycelium allows *A. bisporus* to fruit after it has colonized the casing layer. The fruiting bodies are harvested in 2–3 flushes separated by 7–8-day intervals and a typical yield of 30 kg m^−2^ and a bulk density of 85–95 kg m^−2^. A total of 44, 29, and 8% of cellulose, xylan, and lignin are degraded during the process of colonization of the casing and fruiting body formation when compared to the end of phase III. Based on these data, about 20% of the polysaccharides originally present in the substrate (i.e., before composting) are not consumed. This calculation does not take into account the conversion of substrate in vegetative mycelium (Vos et al. 2017a). Yet, still a significant part of the substrate will have remained intact after growth and fruiting of *A. bisporus.* These plant polysaccharides as well as the vegetative mycelium of *A. bisporus* enable the use of its SMS in various applications.

## Spent mushroom substrate for energy production

SMS can be burnt to produce energy (Zhu et al. 2013). This is neither environment-friendly nor economic because SMS often contains high ash content, which makes the process less effective and leads to new waste problems. As an alternative, SMS can be subjected to combustion, pyrolysis, and gasification (Finney et al. 2009). Combustion is the most efficient process because it is self-sustaining and temperatures are generated that can be used for the production of saleable heat and/or power. Gasification has not been successful, while pyrolysis resulted in a mixture of solid, liquid, and gaseous fuels. However, their calorimetric value was not sufficiently high for wide-scale use of energy production.

Methane was produced using a mixture of dairy manure and SMS of *Flammulina velutipes* or *Pleurotus eryngii*. Methane yield from the mixture was higher than the yield from SMS or dairy manure alone, indicating a synergistic effect of co-digestion (Luo et al. 2018). SMS can also be used as a source of sugars for bioethanol production (Kapu et al. 2012; Zhu et al. 2013). SMS of *A. bisporus* consists of 30% sugar, most of which consists of cellulose and other glucans (19%) and xylan (8%) (Kapu et al. 2012). Chemical and enzymatic hydrolysis released > 40% of the xylose and close to 100% of the glucose. Thus, about 300 mg reducing sugar can be released per gram SMS. SMS of *P. ostreatus* resulting from a substrate consisting of hay, wheat straw, corn cobs, and cotton seed has also been explored as a source of sugars for bioethanol production. This SMS still contains about 40% cellulose and 20% hemicellulose (Zhu et al. 2013). Chemical and enzymatic treatment resulted in about 40% SMS residue and 25% total reducing sugars, most of which being glucose and xylose. One gram of these sugars can be converted to about 0.5 g ethanol (Vieira dos Santos et al. 2016; Gutiérrez-Rivera et al. 2012). Thus, 1 ton of SMS in these examples would yield up to 150 kg of ethanol. In fact, an amount of 187 g ethanol per kg dry matter was produced from *P. ostreatus* SMS derived from sorghum chaff (Ryden et al. 2017).

## Spent mushroom substrate as compost

“Modern” agricultural practice depends heavily on the use of mineral fertilizers. Production of these fertilizers has an enormous environmental impact illustrated by the fact that nitrogen fertilizer production accounts for more than 50% of total energy use in commercial agriculture (Woods et al. 2010). Moreover, inorganic fertilizer may result in decreased nutrient- and water-holding capacities of soils (Mäder et al. 2002), necessitating the use of even more fertilizer. The removal of unsustainable amounts of biomass from fields, for example for biofuel production, increases such adverse environmental effects (Lal 2005). This thus, at least partly, offsets the CO_2_ emissions that are saved by the use of biofuels. SMS could (partly) replace inorganic fertilizer. Mineral fertilizers are superior to SMS with respect to nitrogen, phosphorous, and potassium content. However, nutrient release is slower in the case of SMS and therefore plants can use them more effectively (Uzun 2004). In addition, SMS improves soil structure by increasing organic matter, water capacity, microbial activity, soil temperature, and by decreasing soil compaction. Application of 100 tons SMS per hectare resulted in a 50% increased yield of barley in the same year, being similar to that of inorganic fertilizer. In the case of SMS, this was accompanied by a 2.3-fold increase in soil phosphor and an increase of 40 and 28% of soil organic carbon and nitrogen, respectively, directly after the harvest. In addition, calcium, potassium, and magnesium levels increased up to 3-fold. Notably, inorganic fertilizer did not increase any of these levels (Courtney and Mullen 2008). Thus, SMS can be beneficial for crop yield and soil properties but dosage is important. A 40-ton SMS application per hectare outperforms an 80-ton application in horticultural cucumber production, although both have positive effects (Polat et al. 2009). The high ash content of button SMS (i.e., 50% of dry matter) should also be taken into account. Plants are generally sensitive to salt, while Mg deficiency may result from high SMS application due to antagonism with potassium, which is high in button SMS (Uzun 2004). Ash content can be reduced in button SMS by a weathering process of 6 months, during which SMS spread is in heaps of about 1.5 m height and exposed to the elements (Uzun 2004). Leaching SMS under laboratory conditions with distilled water results in loss of 15% of total nitrogen, 33% of total phosphorus, and 94% of total potassium after 60 days (Guo et al. 2001). Leaching can be reduced by converting SMS to biochar by pyrolysis (Lou et al. 2017). This thus may prevent contamination of groundwater and water bodies when high amounts of SMS are used (Laird et al. 2010; Bradley et al. 2015).

SMS can not only be used in pure form as a fertilizer but can also be used to improve quality of pig manure-based compost (Li et al. 2018). Composting time is reduced when SMS instead of corn stover is used as a bulking agent during composting of pig manure. Moreover, SMS reduces NH_3_ and N_2_O emissions by 36 and 46%, respectively, when compared to corn stover. Although SMS increases CH_4_ emission by 10%, a 34% decrease in global warming potential of CH_4_ and N_2_O is obtained by SMS treatment and is therefore a favorable bulking agent for reducing gaseous emission and increasing compost quality. Similar results have been obtained when SMS is added to a sewage sludge composting (Meng et al. 2018).

SMS can also be used to produce biofertilizer (Zhu et al. 2012). Biofertilizers enhance crop yield thereby promoting sustainable agricultural development. *Pichia farinose* is a stress-tolerant yeast that can be used as a biofertilizer because of its ability to solubilize phosphate. As a result, it improves the growth of soybean. *P. farinose* biofertilizer can be produced on SMS residue and part of the free reducing sugars resulting from chemical and enzymatic-treated SMS of *P. ostreatus*.

## Spent mushroom substrate as mushroom substrate

SMS can be used as a substrate for the production of edible mushrooms. After mushroom production, *L. edodus* leaves 85% of the hemicellulose, 44% of the cellulose, and 77% of the lignin unused (Royse 1992). This SMS can be used for production of *Pleurotus sajor-caju* mushrooms (Royse 1992) but a 20% higher production is obtained by supplementing 10% wheat bran and 10% millet. Similarly, button mushrooms of *Agaricus blazei* can be produced on straw-based SMS from oyster mushroom cultivation complemented with 20% vermi-compost or sunflower seed hulls (González Matute et al. 2011). These production levels are similar when compared to standard compost.

## Spent mushroom substrate as animal feed

The European Union (EU) depends for 70% on the import of protein-rich animal feed, mainly based on soya. The EU aims to reduce this import dependency and is therefore looking for alternatives like insect protein. This makes sense considering the fact that flies are a natural food source for pigs, poultry, and many fish species. Moreover, insects are very efficient in converting feed into body mass, and they emit fewer greenhouse gases and less ammonia than cattle and pigs. In addition, they require less land and water than cattle. At the moment, insect protein can be used to feed fish. Legislation is under way to allow this food source also for poultry. SMS is an interesting feed source for insects. Yet, this has so far received little attention, even though they are the largest group of fungivores in nature. Many insects even depend on mycelium or mushrooms within their life cycle (Vega and Blackwell 2005). For instance, 136 taxa of beetles from 30 different families are associated to mushrooms of *P. ostreatus*, about 60% of which being obligate fungivores (Cline and Leschen 2005). Larvae also develop within the mushrooms, especially from the families of Erotylidae and Mycetophagidae.

SMS may also be used to directly feed fish, poultry, pigs, and cows. Several studies report the use of mushrooms or mushroom-extracts as feed. It is tempting to speculate that these results can be extrapolated to SMS. Fingerlings of *Labeo rohita* and *Hemigrammus caudovittatus* were fed with a diet of 9% fish meal and 9% mushroom meal, 9% fishmeal and 9% worm meal, or 18% fish meal. The diet with earth worm meal showed approximately 2-fold higher growth rate when compared to the fish meal diet, while the diet with mushroom showed a 1.2–1.7-fold increase. These data indicate that both earthworm meal and mushroom can be a supplement in fish diet, reducing the need for fish meal (Paripuranam et al. 2011). In addition, a mushroom-based diet can stimulate the immune response of fish. Supplementation of 2% shiitake mushroom extract in the diet of the rainbow trout *Oncorhynchus mykiss* improved immunological parameters and survival rate of the fish when exposed to the bacterial pathogen *Lactococcus garvieae* (Baba et al. 2015)*.* Similarly, shiitake extracts had positive effects on health parameters of chicken but they did not promote growth (Willis et al. 2004).

Supplementation of pig feed with ≥ 5% of a fermented mixture of *P. ostreatus* SMS with rice and barley bran shows negative effects on weight gain, while 3% supplementation had no effect (Song et al. 2007). Feeding trials with cows show contrasting results. Cows only consume a mixture of ≤ 17% straw-based *P. ostreatus* SMS in a basic feed of hay and maize silage (Adamovic et al. 1998). This 17% supplementation impacts weight gain compared to the control and 10% SMS supplementation. This is surprising considering experimental results that suggest that *Pleurotus sajor-caju* and *P. ostreatus* improve the digestibility of straw due to the degradation of lignin and cellulose (Adamovic et al. 1998). The improved digestibility of straw may be counteracted by the presence of fungal mycelium. A positive effect on growth was observed by fermenting SMS with lactic acid bacteria. Supplementation with 10% fermented sawdust-based *P. ostreatus* SMS improved growth performance of post-weaving calves by 8% (Kim et al. 2011).

## Spent mushroom substrate for materials

Large-scale use of plastics made from non-renewable resources like crude oil and natural gas dates back to ~ 1950 (Geyer et al. 2017). The largest market for plastics is packaging. Its share in municipal solid waste increased from 1% in 1960 to > 10% by 2005 in middle- and high-income countries (Jambeck et al. 2015). These plastics slowly degrade resulting in their accumulation in landfills and the natural environment (Barnes et al. 2009). There is a need for alternatives for synthetic materials with diminishing oil reserves, increasing fuel prices, and global mitigation against climate change and environmental damage (Jones et al. 2017). Mycelium materials, i.e., mycelium composites and pure mycelium, are such alternatives. Mushroom-forming fungi seem particularly interesting to produce such materials because of their capacity to form large networks (Ferguson et al. 2003) and the fact that they can efficiently colonize lignocellulosic material such as that in low-quality organic waste streams like saw dust and straw. Mycelium composites consist of a network of fungal hyphae binding together the particles within the substrate that represent the bulk of the material. Properties of composite mycelium materials like compressive strength, flexibility, and electrical conductivity depend on the fungal species used, the feedstock, additives, and environmental growth conditions (Jones et al. 2017). In general, mycelium composites have properties similar to those of polymer foams. They can be used as packaging material (Holt et al. 2012) or in construction (Xing et al. 2018), while various other applications have been proposed such as acoustic dampers, absorbents, paper, textiles, and vehicle and electronic parts (Jones et al. 2017). In the next few years, the full potential of mycelium composite materials will be unveiled. This will also include the potential of SMS as a starting material to produce mycelium composites.

Production of mycelium composites includes inactivation of the fungus for instance by a heat treatment. The time before inactivation determines the ratio between plant and fungal biomass. The fungus is inactivated at some point during colonization to generate a mycelium composite material. Prolonging growth will ultimately result in a pure mycelium material that also shows interesting properties (Islam et al. 2017; Haneef et al. 2017; Appels et al. 2018) being impacted by the fungal species and the substrate used. Growth on cellulose and cellulose/potato-dextrose results in a stiffer material in the case of *P. ostreatus* when compared to that *Ganoderma lucidum.* In addition, dextrose-containing substrates results in materials of these fungi that are more elastic. Light and CO_2_ also impact material properties (Appels et al. 2018). The maximum tensile strength of mycelium ranges between 5.1 and 9.6 MPa when the wild-type strain is grown in the dark or in the light at low or high CO_2_ levels. Notably, a strain in which the hydrophobin gene *sc3* is inactivated (van Wetter et al. 2000) forms a mycelium material with a 3–4-fold higher maximum tensile strength due to increased mycelium density (Appels et al. 2018). Apart from its effect on mycelium density, *sc3* is also involved in surface hydrophobicity of the mycelium (van der Vegt et al. 1996; Wösten et al. 1993, 1994, 1999). Indeed, the *sc3* deletion strain retains more water when compared to the wild type. Together, it is concluded that genetic modification and environmental growth conditions impact properties of the mycelium. In fact, mechanical properties of wild-type mycelium were similar to those of natural materials, while those of the *sc3* deletion strain were more similar to thermoplastics. Future research should unveil whether it is possible to convert SMS to a pure mycelium. Clearly, full degradation of the substrate requires much more time and results in a larger weight loss of the material when compared to the decomposition needed to produce mycelium composites. As such, pure mycelia are more expensive to produce.

## Isolation of enzymes and bioactive molecules from spent mushroom substrate

Lignocellulosic enzymes such as laccase, lignin peroxidase, cellulase, and xylanase are secreted by mushroom-forming fungi to degrade their substrate into molecules that can be taken up to serve as nutrients. These enzymes can be extracted from SMS for applications like biofuel and biogas production (Phan and Sabaratnam 2012; Wan and Li 2012). Compared to physical and chemical pre-treatments of biofuel feed stocks, a fungal pre-treatment of lignocellulosic materials for the production of second-generation biofuels is environmentally friendly and energy efficient (Wan and Li 2012). However, production costs of enzymes have been calculated to be between US$0.6 and 1.3 per gallon bioethanol (Klein-Marcuschamer et al. 2012), representing 40–87% of the sales price of the biofuel. This major cost for bioethanol production could be decreased by reducing fermentation time and the complexity of the enzyme production process (Klein-Marcuschamer et al. 2012). Enzymes present in SMS could be a good alternative because there is no fermentation time needed, and the extraction process can be low tech. For instance, enzymes can be simply extracted from SMS with water (Mayolo-Deloisa et al. 2009; Jurak et al. 2015a; Vos et al. 2017b). Such extracts may be used directly, either or not mixed with enzymes from other sources, to convert for instance lignocellulosic waste streams in sugars for second-generation biofuels. They may also be (partly) purified from the extract by a simple two-phase separation using a poly-ethylene glycol salt system. For instance, 95% of laccase activity could be recovered using such a system (Mayolo-Deloisa et al. 2009). Future research should assess which types of SMS show the best enzyme activities for particular lignocellulosic feed stocks. The crude SMS enzyme extracts could also be used for purification of specific enzymes used in technical applications or in food and feed. The activities that are extracted will determine whether this will be commercially viable.

## Conclusions

**Fig. 1:**
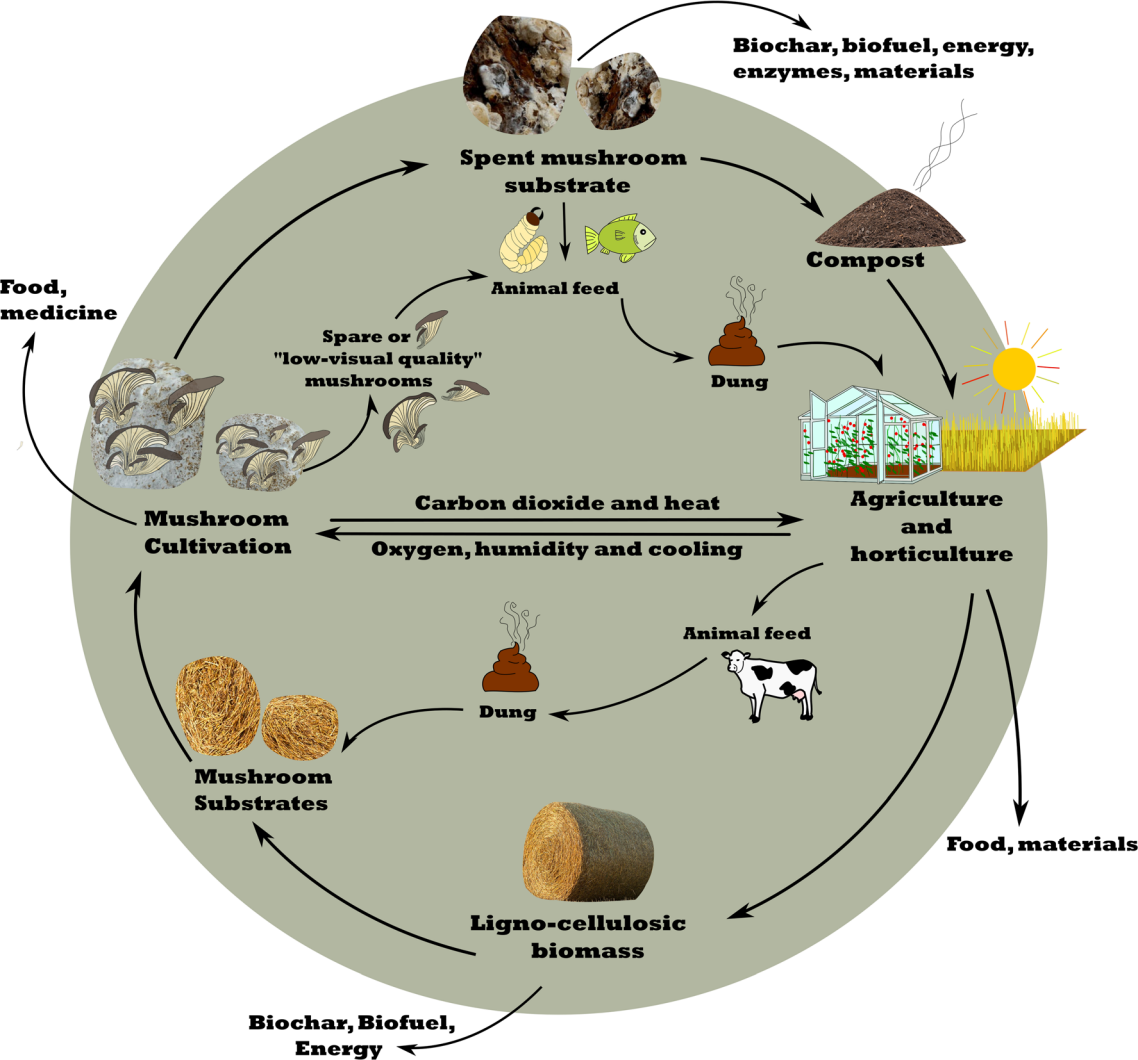
Use of SMS in a circular economy

Global mushroom production has increased rapidly last decades and is expected to further increase in the future due to the need for more high-quality food with a reduced environmental impact. This will be accompanied by an increase in SMS, which may exceed a trillion kg a year, representing 6 tons of SMS km^−2^ global land area. There are different options to use these enormous amounts of SMS (Fig. 1), but the question is how it can be used in the most circular way. Intuitively, one would start with extracting enzymes from the spent substrate. Whether this will be commercially viable depends on the activities present in SMS and the energy and water needed for extraction, and, if needed, for purification. After enzyme extraction, SMS could be used for one or two other rounds of mushroom production, followed by using the SMS as compost, feed, or a source for biofuel production. The most circular option will depend on geographical location, being for instance dependent on the local presence of waste streams, fertilizers, and food and feed resources. Future research should be dedicated to calculate which (order of) applications of SMS are truly environmentally friendly and economical viable and how this could be improved by integrating mushroom and SMS production within the agri- and horticultural system. For instance, waste stream of greenhouses could be used for mushroom and SMS production, while the CO_2_ and heat resulting from these processes could be used to stimulate plant growth in green houses. The impact can be high since doubling the atmospheric CO_2_ content in greenhouses increases plant growth by 33% (Kimball 1983). Question is whether mushroom production facilities should be concentrated or spread within geographical areas. Concentration of mushroom production could lead to environmental problems (Leiva et al. 2015) but could also supply sufficient SMS to make applications economical and environmentally viable. Together, SMS has great potential in a circular economy but we are in great need for quantitative models predicting environmental impact and economic viability.

## Data Availability

Not applicable.
